# Threshold for basophil activation test positivity in neuromuscular blocking agents hypersensitivity reactions

**DOI:** 10.1186/1710-1492-9-42

**Published:** 2013-10-23

**Authors:** Natalia Hagau, Nadia Gherman-Ionica, Manuela Sfichi, Cristina Petrisor

**Affiliations:** 1 Department of Anaesthesia and Intensive Care, University of Medicine and Pharmacy "Iuliu Hatieganu", Cluj-Napoca, Romania; 2 Department of Allergology and Immunology, University of Medicine and Pharmacy "Iuliu Hatieganu", Cluj-Napoca, Romania; 3 Department of Immunopathology, Clinical Emergency County Hospital Cluj, Cluj-Napoca, Romania; 4 Department of Anaesthesia and Intensive Care, Clinical Emergency County Hospital of Cluj, 400006 Clinicilor 3-5, Cluj-Napoca, Romania

**Keywords:** Basophils, Drug hypersensitivity, Flow cytometry, Neuromuscular blocking agents

## Abstract

**Background:**

Several different criteria for the positivity of the flow-assisted Basophil Activation Test (BAT) for the diagnosis of Neuromuscular Blocking Agents (NMBA) hypersensitivity reactions have been used in past studies. Our aims were to determine the threshold for BAT positivity expressed as the stimulation index (SI, calculated as the percentage of activated basophils after stimulation with NMBA divided by the number of basophils with no NMBA stimulation) and as the percentage of activated basophils, and to determine the sensitivity and specificity of BAT for NMBAs.

**Methods:**

22 consenting adult patients with previous intraanaesthetic NMBAs-related hypersensitivity reactions were tested for the culprit drug. 34 controls who tolerated NMBAs were similarly tested. BAT was performed using Flow2Cast technique and the up-regulation of the CD63 marker on the basophils was measured using Cell Quest programme (FACSCalibur Becton Dickinson, USA). Receiver operating characteristics curve (ROC) analysis was performed.

**Results:**

ROC curve analysis for BAT results versus history yields a stimulation index of 1.76 as the optimal threshold, with an AUC of 0.81 (CI 95% 0.69-0.93, p < 0.01) and a percentage of activated basophils > 5.01%, with an AUC of 0.84 (CI 95% 0.72-0.95, p < 0.01). Considering both thresholds (the SI ≥ 1.76 together with the percentage of activated basophils > 5%) as diagnostic criteria, 15 patients had positive BAT, the overall BAT sensitivity being 68.18% (CI 95% 45.11-82.26%). None of the controls fulfilled both criteria and the specificity of the test was 100% (CI 95% 87.35-100%).

**Conclusions:**

With a stimulation index ≥ 1.76 and a percentage of activated basophils > 5.01% as threshold, the performance of BAT for NMBAs yields 68.18% sensitivity and 100% specificity.

## Background

Neuromuscular blocking agents (NMBAs) represent the most common cause of adverse reactions during anaesthesia, including immediate-type hypersensitivity reactions [1-3]. The diagnosis of intraanaesthetic anaphylaxis implies the retrospective determination of the responsible agent and of the cross-reactive compounds [4]. Skin tests coupled with history remain the mainstay of the diagnosis of an IgE-mediated reaction, but though reliable, they are not infallible [5].

The quantification of basophil activation by flow cytometry (the basophil activation test, BAT) has been proven to be a useful tool for the assessment of the immediate-type response to allergens mediated by IgE or by other mechanisms in drug allergic patients [6]. There are still open questions regarding the threshold for positivity [7]. Several different criteria and cut-offs have been used for BAT with NMBAs in previous studies, with a percentage of activated basophils, after stimulation with the allergen, ranging from 4% to 15% or an increase in the percentage of basophils of more than 10 or 15% above the percentage of spontaneously activated basophils [1,8-11]. Moreover, the cut-off for BAT positivity expressed as a stimulation index (the percentage of activated basophils after stimulation with the culprit NMBA divided by the percentage of activated basophils with no stimulation) has not yet been established.

The aims of our study were to determine the threshold for BAT positivity expressed as the stimulation index and as the percentage of activated basophils and to determine the sensitivity and specificity of BAT for NMBAs using Flow2CAST technique.

## Methods

### Subjects

Ethical approval for this study (Ethical Committee N° 6/2008) was provided by the Research Ethics Committee of the University of Medicine and Pharmacy "Iuliu Hatieganu" Cluj-Napoca (Chairman Prof. Felicia Loghin) on 2nd Apr 2008. After obtaining patients’ informed consent, a total of 66 patients with previous general anaesthesia (in which NMBAs were administered) and intraanaesthetic drug allergy referred to our clinic were tested in our allergoanaesthesia department.

The inclusion criteria was history suggestive of an immediate-type hypersensitivity reaction to NMBAs and positive skin tests. From the 66 patients, 22 consenting adult patients with previous intraaneaesthetic NMBA-related allergy suspected by the attending anaesthesiologist and positive skin tests for the culprit drug were included.

The exclusion criteria were: 23 patients with signs or symptoms which were not specific for drug hypersensitivity and had negative skin tests, 9 patients with other drug allergies (3 midazolam, 4 antibiotics, 2 propofol), one patient with age less than 18 years, and also, 5 patients with suspected NMBAs hypersensitivity reactions having negative skin tests.

The control group included: 34 patients without previous drug allergies who underwent surgery and tolerated intravenous NMBAs in our hospital, who were similarly tested *in vivo* and *in vitro* tests for the NMBAs they received during surgery.

### Skin tests

*In vivo* tests, the skin prick test (SPT) and the intradermal test (IDT), were performed using commercially available solutions of atracurium, pancuronium, rocuronium and suxamethonium by an allergologist experienced in skin testing. Normal saline solution (0.9% NaCl) was used to dilute the commercial substances. The concentrations used were established as being the maximal non-reactive concentrations [4,12]. The SPTs and IDTs were performed in conformity with international recommendations [13]. We used 1% histamine as the positive control and NaCl 0.9% as the negative control. The SPT was considered positive when the wheal diameter was superior to 3 mm within 20 minutes. For IDT the wheal area was marked initially and 20 minutes after testing. An increase in diameter greater than 3 mm or the doubling of the initial injection wheal represented a positive result [14].

### Flow cytometry- the basophil activation test

Flow cytometric analysis of *in vitro* activated basophils was performed with Flow2Cast technique, which uses CCR3 as the basophil identification marker and CD63 as the marker of basophil activation (Bühlmann Laboratories AG, Switzerland). The immunologist was blinded regarding the patients’ or controls’ history and skin test results. Flow cytometry was performed on the same day with skin testing using all four tested NMBAs for each patient. We used 6 test tubes containing 50 μl of whole blood. The blood was collected into K-EDTA venipuncture tubes, up to the dedicated volume, from antecubital vein (no garrot). We performed the cell stimulation immediately after collection of the blood and we did not store the blood samples. The first sample was mixed with 50 μl of stimulation buffer as negative control. The next two samples were mixed with 50 μl solution of anti-FcεRI (a highly specific monoclonal antibody for the IgE receptor) and 50 μl solution of FMLP (an unspecific cell activator- the chemotactic peptide N-Formyl- Met-Leu), as positive controls. A positive control higher than 10% basophils indicates that the patient is not a nonresponder and excludes CCR3 down-regulation. In the remaining 3 test tubes, 50 μl of NMBAs solution was added. The tested drug concentrations were c1, c2 and c3 for atracurium, rocuronium, suxamethonium and pancuronium (Table 1).

**Table 1:** **NMBA concentrations for *in vivo *** **and *in vitro *** **tests**

**NMBA**	**SPT (mg/mL)**	**IDT (μg/mL)**	**BAT**	**Concentration (μg/mL)**
**Pancuronium**	2	200	c1	500
			c2	50
			c3	5
**Rocuronium**	10	50	c1	500
			c2	50
			c3	5
**Suxamethonium**	10	100	c1	5
			c2	0.5
			c3	0.05
**Atracurium**	1	10	c1	2.5
			c2	0.25
			c3	0.025

SPT = Skin prick test; IDT = Intradermal test; BAT = Basophil activation test.

Subsequently, 20 μl staining reagent with two monoclonal antibodies, anti- CCR3-PE (human chemokine receptor labelled with phycoerythrin) and anti-CD63-FITC (or Gp53, a glycoprotein expressed on activated basophils), were added in each tube. The samples were incubated for 15 min at 37°C in a water bath. A prewarmed lysing solution of 2 ml was added to each tube and incubated for 10 minutes at room temperature. After centrifuging (500 × g, 5 minutes) and washing, the cells were suspended in 300 μl wash buffer. Our laboratory limit of basophilic cells analyzed for allergies was set to 500.

On our histogram defined by forward scatter and side scatter, several populations of cells are identified: CCR3-positive cells (basophils and eosinophils, the main effector cells in allergic inflammation) and CCR3-negative cells (lymphocytes, monocytes and granulocytes) [15]. Basophils are characterised as being the brightest cells (showing high-density fluorescence with anti-CCR3-PE label) and having low side-scatter.

The up-regulation of CD63 marker on the basophils was measured using Cell Quest programme (FACSCalibur Becton Dickinson San Jose California USA Analyzer 2001).

### Statistical analysis

The stimulation index (SI) for all subjects and all concentrations was analysed, as well as the percentage of activated basophils after stimulation with the culprit NMBA. The stimulation index is calculated as the percentage of activated basophils after stimulation with NMBA divided by the percentage of activated basophils with no NMBA stimulation. Receiver operating characteristics curve (ROC) analysis was performed with SI and the percentage of activated basophils as discrimination variables. The reference standard was considered when the patients had positive history of allergy to NMBA. The area under curve (AUC) and p-values were evaluated [16,17]. The cut-off in the ROC curve is the closest point on the ROC curve to the point (0,1) which is the point of absolute classification, where sensitivity and specificity are 1 [18]. For better prediction, it is obvious to see if using the highest SI from all three NMBA concentrations for each drug and for each subject we could obtain an optimal SI as classification variable. We performed the ROC curve analysis for the highest stimulation indexes and for the highest percentage of activated basophils for all three NMBA concentrations in patients and controls to calculate the optimal cut-off value. Sensitivity was calculated as the number of patients with positive BAT divided by the total number of patients, while specificity was calculated as the number of controls with negative BAT divided by the total number of controls.

The result of BAT was considered positive when at least one of the SI or percentage of activated basophils for c1, c2 or c3 (the highest stimulation index) was higher than the threshold.

## Results

A total of 22 patients with an immediate intraanesthetic hypersensitivity reaction caused by NMBAs were tested *in vivo* and *in vitro*, as well as 34 surgical patients as controls, between January 2008-December 2012. Controls were administered the tested NMBAs intraoperatively without showing any signs of intraanesthetic drug allergy. Rocuronium was the culprit agent in 8 patients, atracurium in 11, suxamethonium in 1 and pancuronium in 2 patients. Clinical symptoms of NMBA allergy were bronchospasm in 3, angioedema in 2, urticaria in 4, hypotension in 2 and anaphylactic shock in 11 patients (Table 2). All patients presented positive skin tests. None of the healthy controls presented a positive skin test (Table 3). None of the subjects presented systemic reactions during the skin tests.

**Table 2:** Positive history patients’ data

**Number**	**Sex**	**Substance**	**Clinical symptoms**	**SPT**	**IDT**	**SI**	**Ba%**
1	M	Rocuronium	Shock	Positive		6.06	10.73
2	M	Suxamethonium	Shock	Positive		2.10	31.56
3	M	Pancuronium	Shock	Negative	Positive	1.06	7.15
4	M	Rocuronium	Urticaria	Positive		2.60	13.00
5	F	Pancuronium	Bronchospasm	Negative	Positive	3.23	17.11
6	F	Rocuronium	Shock	Positive		2.05	14.48
7	F	Atracurium	Shock	Positive		0.93	2.17
8	F	Rocuronium	Shock	Negative	Positive	5.22	25.15
9	F	Atracurium	Shock	Positive		7.68	20.50
10	F	Atracurium	Angioedema	Negative	Positive	1.86	3.54
11	F	Atracurium	Angioedema	Positive		1.99	13.29
12	F	Atracurium	Shock	Positive		4.81	7.93
13	F	Atracurium	Urticaria	Positive		3.89	8.91
14	F	Atracurium	Hypotension	Negative	Positive	2.01	6.48
15	F	Rocuronium	Hypotension	Positive		0.52	2.14
16	M	Rocuronium	Urticaria	Positive		4.62	18.68
17	M	Atracurium	Bronchospasm	Negative	Positive	9.11	20.67
18	F	Rocuronium	Bronchospasm	Positive		1.60	7.19
19	F	Atracurium	Shock	Negative	Positive	5.03	5.08
20	F	Atracurium	Shock	Negative	Positive	2.62	3.54
21	F	Rocuronium	Urticaria	Negative	Positive	4.82	10.26
22	F	Atracurium	Shock	Negative	Positive	1.48	2.67

M = Masculine; F = Feminine; Ba% = The highest percentage of activated basophils from c1, c2 and c3 concentrations for each NMBA; SPT = Skin prick test; IDT = Intradermal test; SI = The highest stimulation index from c1, c2 and c3 concentrations for each NMBA.

**Table 3:** Controls’ data

**Number**	**Sex**	**Substance**	**SI**	**Ba%**
1	M	Suxamethonium	1.15	5.01
2	M	Atracurium	1.57	4.00
3	M	Pancuronium	1.71	2.09
4	M	Pancuronium	1.90	3.90
5	M	Suxamethonium	1.04	2.72
6	M	Atracurium	1.51	3.09
7	M	Rocuronium	1.87	4.54
8	M	Atracurium	1.33	3.22
9	M	Pancuronium	1.36	3.76
10	M	Rocuronium	1.49	3.02
11	M	Atracurium	0.83	1.30
12	M	Pancuronium	1.76	3.60
13	M	Pancuronium	1.67	3.40
14	F	Pancuronium	1.03	3.32
15	F	Atracurium	1.63	3.93
16	F	Rocuronium	1.69	3.49
17	F	Rocuronium	1.33	6.70
18	F	Suxamethonium	1.19	6.00
19	F	Pancuronium	1.13	4.00
20	F	Rocuronium	1.38	5.59
21	F	Suxamethonium	1.02	1.80
22	F	Pancuronium	1.05	4.00
23	F	Rocuronium	0.85	3.25
24	F	Suxamethonium	1.37	4.05
25	F	Atracurium	2.92	3.68
26	F	Rocuronium	1.21	2.96
27	F	Suxamethonium	1.32	3.23
28	F	Atracurium	1.29	3.16
29	F	Suxamethonium	1.99	3.14
30	F	Atracurium	2.24	3.59
31	F	Rocuronium	1.23	5.75
32	F	Pancuronium	1.55	0.59
33	F	Rocuronium	1.76	0.67
34	F	Atracurium	2.29	0.87

M = Masculine; F = Feminine; Ba% = The highest percentage of activated basophils from c1, c2 and c3 concentrations for each NMBA; SI = The highest stimulation index from c1, c2 and c3 concentrations for each NMBA.

**Figure 1:**
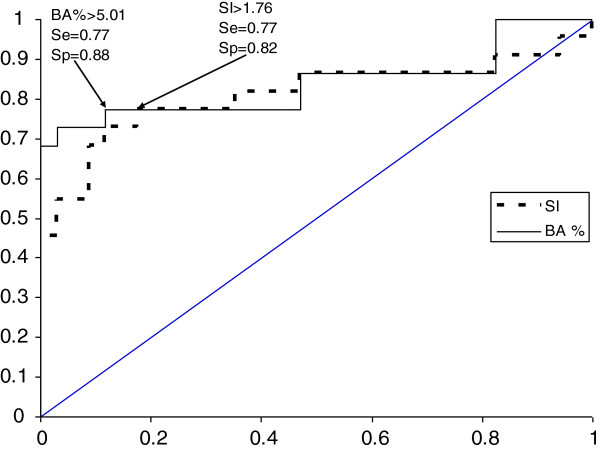
**ROC curve for BAT stimulation index (SI) and percentage of activated basophils (Ba%) versus history for patients and controls.** Ba% = the highest percentage of activated basophils from c1, c2 and c3 concentrations for each NMBA; SI = the highest stimulation index from c1, c2 and c3 concentrations for each NMBA; Se = sensitivity; Sp = specificity.

ROC curve analysis for BAT performed with *the highest stimulation index* from the three NMBAs concentrations used together versus history yields a stimulation index of 1.76 as the optimal threshold for BAT positivity, with an AUC of 0.81 (CI 95% 0.69-0.93, p < 0.01) (Figure 1). With a cut-off of 1.76 as diagnostic criteria, there were 17 positive BAT in patients and 6 positive BAT for controls, thus the sensitivity of BAT was 77.27% (CI 95% 54.17-91.31%) and the specificity 82.35% (CI 95% 64.83-92.61%) when using the optimal SI generated by the ROC curve as diagnostic criteria alone. For rocuronium, from the 8 patients 6 were BAT positive, with 75% sensitivity. From the 11 patients with atracurium-induced anaphylaxis 9 had positive BAT, with 81.81% sensitivity.

**Figure 2:**
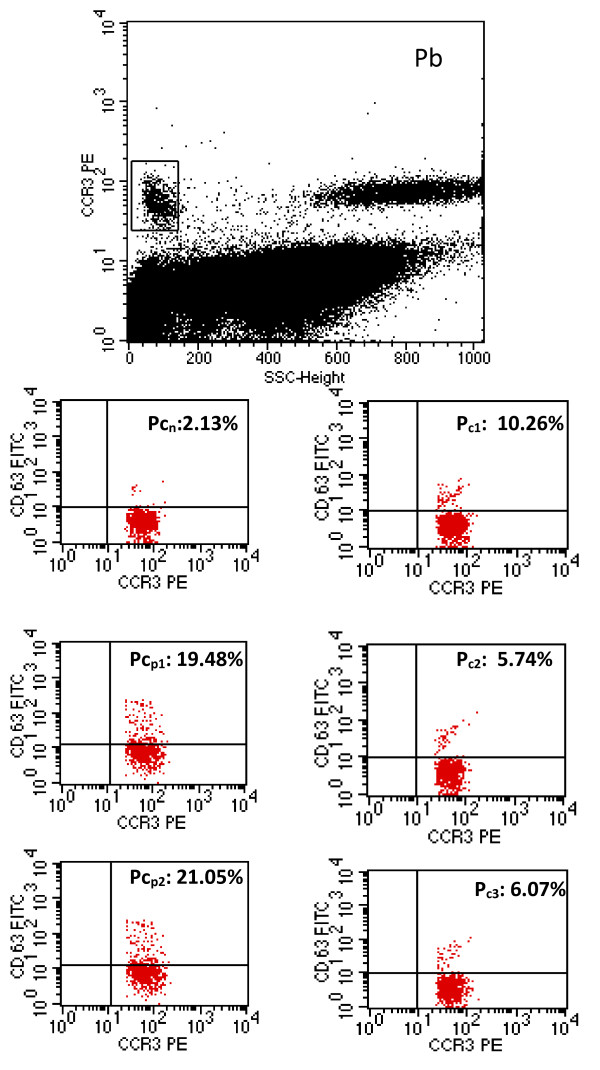
**Flow cytometry dot plot result in a patient with rocuronium-induced anaphylactic shock.** Pb = initial plot identifying basophils; Pc_n_ = negative control; Pc_p1_ = positive control with FcεRI; Pc_p2_ = positive control with N-formyl-Met-Leu; P_c1_ = allergen rocuronium 500 μg/mL; P_c2_ = allergen rocuronium 50 μg/mL; P_c3_ = allergen rocuronium 5 μg/mL.

In order to find the optimal threshold expressed as *the percentage of activated basophils*, we performed the ROC curve using the highest Ba% value for BAT from the three drug concentrations for each patient (Figure 1). The optimal threshold value for BAT positivity is a percentage of activated basophils > 5.01%, with an AUC of 0.84 (CI 95% 0.72-0.95, p < 0.01), as revealed by performing the ROC curve. Considering Ba% > 5.01% as the threshold for BAT positivity, there were 17 BAT positivities among the 22 patients and 5 out of the 34 controls had positive BAT, with an overall 77.27% (CI 95% 54.17-91.31%) BAT sensitivity and 85.29% (CI 95% 68.16-94.45%) specificity. Three patients were BAT positive only considering a percentage of activated basophils > 5.01%. From the 8 allergic reactions caused by rocuronium, 7 were confirmed by a positive BAT (87.50% sensitivity), for atracurium BAT confirmed 7 out of the 11 allergic reactions (63.63% sensitivity). Figure 2 provides the flow cytometry dot polt for one of the patients with rocuronium-induced anaphylactic shock, showing positive results for all three rocuronium concentrations.

The use of a SI ≥ 1.76 or a Ba% > 5.01% in the interpretation of BAT results shows no differences between the AUC (p = 0.69).

Considering *both thresholds* obtained from the ROC curves (the SI ≥ 1.76 together with the percentage of activated basophils > 5%) as diagnostic criteria, 15 patients had positive BAT. Thus, the overall BAT sensitivity performed with the three NMBAs concentrations was 68.18% (CI 95% 45.11-85.26%). None of the controls fulfilled both criteria and the specificity of the test was 100% (CI 95% 87.35-100%).

## Discussion

NMBAs-induced anaphylaxis is a rare intraanesthetic event having an estimated incidence of 250.9/ 1 million anaesthetics, with life-threatening clinical symptoms like hypotension, shock or cardiac arrest in 36.39% cases of non-IgE-mediated reactions and 84.04% IgE-mediated allergic reactions [3].

The diagnostic management of anaphylaxis from NMBAs rests upon an evocative history corroborated by appropiate skin tests, which are reliable but have no absolute diagnostic accuracy [4]. For example, when the skin test is negative, it is virtually impossible to determine whether it is a false-negative test unless the agent is administered [19]. However, the performance of challenge tests for NMBAs is restricted because of ethical and practical limitations [20]. For NMBAs, allergological skin tests still remain the current reference test [8].

Basophil activation based upon the expression of CD63 in the presence of specific allergens was found to be of importance for the diagnosis of IgE-mediated hypersensitivity [1]. We chose to perform BAT using CD63 as activation marker as this seems to be the most sensitive for NMBAs [11,21]. In previous studies the sensitivity of BAT for NMBAs was 36-92%, while the specificity was 93-100%, depending on the chosen threshold for positivity [1,8-11].

There are still many open questions regarding the most appropiate threshold for positivity. In the past, thresholds for positivity often were set arbitrarily [7]. For other drugs, the threshold expressed as a stimulation index ≥2 has been established by ROC curve analysis. A SI ≥ 2 together with a percentage of activated basophils of more than 5% (in order to avoid small, un-specific stimulation) represent current criteria for BAT positivity for antibiotics and non-steroidal anti-inflammatory drugs [22-24]. These criteria are also recommended by the manufacturers for NMBAs, though for NMBAs the threshold expressed as a stimulation index has not been established by the performance of ROC curves including a large number of patients.

Optimal allergen specific thresholds should be used, instead predefined and arbitrarily chosen cut-offs, and these are adequately defined by performing receiver operating characteristics (ROC) curves [25]. The ideal way to choose a cut-off for a given test is by ROC curve analysis [26]. This enables the precise calculation of the most discriminative cut-off value [27]. The cut-off for BAT positivity expressed as stimulation indexes and established by the use of ROC curve analysis has not been assessed in most published studies on NMBAs [1,9-11]. The only published ROC curve for NMBAs included 14 patients with rocuronium-induced anaphylaxis and 8 healthy controls and a sensitive cut-off of 4% activated basophils has been suggested for rocuronium [8].

The inclusion of sufficient numbers of well characterised allergic patients and healthy, exposed control individuals is critical to perform ROC curve analysis [20]. In our study, 22 patients with intraanesthetic immediate-type allergic reactions after the administration of NMBAs and positive skin tests for the culprit drug on subsequent testing, as well as 34 healthy controls, were included. All controls presented negative skin test for NMBAs and tolerated them without signs of hypersensitivity. The optimal threshold generated by the ROC curve analysis was 1.76 when the stimulation index was analysed and 5.01% when the percentage of activated basophils was considered.

Anaphylaxis from NMBAs can be life-threatening and it is critical to establish a sensitive cut-off as false negative results can have dramatic consequences [8,20].

Using both criteria for diagnosis, similarly to antibiotic and anti-inflammatory drugs and according to the manufacturers’ recommendations, BAT has 68.18% sensitivity and 100% specificity for NMBAs. The lower observed BAT sensitivity might be due to several reasons. First, NMBAs sensitivity might be lost in time [1]. Second, basophil activation is not directly comparable to mast cell reactivity, the letter being the major effector cells in some of the hypersensitive patients [11]. Moreover, false negative results might be explained by a recent exposure to cross-reactive compounds with similar epitopes and the subsequent transient refractoriness of the cells [28].

Our data suggest that BAT for NMBAs might have different sensitivities for each NMBA, but the limited number of cases for each drug does not allow us to draw a definitive conclusion. A higher number of cases are needed in order to increase the power of the study, a target difficult to obtain as anaphylaxis from NMBAs is a low-prevalence disease. The inclusion of a higher number of patients by performing multicenter studies might overcome this limitation of our study.

## Conclusion

With a stimulation index ≥1.76 and a percentage of activated basophils > 5.01% as threshold, the performance of BAT for NMBAs yields 68.18% sensitivity and 100% specificity.

## Abbreviations

BAT: Basophil activation test; NMBA: Neuromuscular blocking agents; SI: Stimulation index (calculated as the percentage of activated basophils after stimulation with NMBA divided by the number of basophils with no NMBA stimulation); ROC: Receiver operating characteristics curve; SPT: The skin prick test; IDT: The intradermal test.

## Competing interests

The authors declare that they have no competing interests.

## Authors’ contributions

NH designed the present study, contributed to the acquisition of data, the analysis and the interpretation of data, and has been involved in drafting the manuscript. NGI performed the allergological tests and MS performed the *in vitro* tests, both have participated in data acquisition and revised the manuscript. CP has participated in data acquisition, data analysis and interpretation and has been involved in drafting the manuscript. All authors have read and approved the final manuscript.
